# The Establishment of a Mouse Model of Recurrent Primary Dysmenorrhea

**DOI:** 10.3390/ijms23116128

**Published:** 2022-05-30

**Authors:** Fang Hong, Guiyan He, Manqi Zhang, Boyang Yu, Chengzhi Chai

**Affiliations:** 1 Jiangsu Provincial Key Laboratory of TCM Evaluation and Translational Research, School of Traditional Chinese Pharmacy, China Pharmaceutical University, Nanjing 211198, China; hongfang0221@126.com (F.H.); heguiyan0429@163.com (G.H.); 2 Department of Medicine, Duke University, Durham, NC 27708, USA; manqi.zhang@duke.edu; 3 Research Center for Traceability and Standardization of TCMs, School of Traditional Chinese Pharmacy, China Pharmaceutical University, Nanjing 211198, China

**Keywords:** primary dysmenorrhea, recurrent, mice model, two estrous cycles, writhing reaction, PGF_2α_, PGE_2_, COX-2, uterine artery blood flow, metabonomic analysis

## Abstract

Primary dysmenorrhea is one of the most common reasons for gynecologic visits, but due to the lack of suitable animal models, the pathologic mechanisms and related drug development are limited. Herein, we establish a new mouse model which can mimic the periodic occurrence of primary dysmenorrhea to solve this problem. Non-pregnant female mice were pretreated with estradiol benzoate for 3 consecutive days. After that, mice were injected with oxytocin to simulate menstrual pain on the 4th, 8th, 12th, and 16th days (four estrus cycles). Assessment of the cumulative writhing score, uterine tissue morphology, and uterine artery blood flow and biochemical analysis were performed at each time point. Oxytocin injection induced an equally severe writhing reaction and increased PGF_2α_ accompanied with upregulated expression of COX-2 on the 4th and 8th days. In addition, decreased uterine artery blood flow but increased resistive index (RI) and pulsatility index (PI) were also observed. Furthermore, the metabolomics analysis results indicated that arachidonic acid metabolism; linoleic acid metabolism; glycerophospholipid metabolism; valine, leucine, and isoleucine biosynthesis; alpha-linolenic acid metabolism; and biosynthesis of unsaturated fatty acids might play important roles in the recurrence of primary dysmenorrhea. This new mouse model is able to mimic the clinical characteristics of primary dysmenorrhea for up to two estrous cycles.

## 1. Introduction

Dysmenorrhea, defined as painful menstrual cramps of uterine origin, is the most common gynecological disorder in women [1,2,3]. The reported prevalence of dysmenorrhea is more than 50% [4,5,6]. Based on pathophysiology, dysmenorrhea is divided into primary dysmenorrhea (PD) and secondary dysmenorrhea [2]. PD refers to cyclic menstrual pain in the absence of pelvic anomalies [2,7,8] while secondary dysmenorrhea is caused by identifiable pathological conditions, such as adenomyosis, endometriosis, fibroids, and pelvic inflammatory disease [2,4,9].

Many women are lacking medical treatment because menstrual pain is usually regarded as a concomitant menstrual symptom [4]. However, PD not only affects the quality of life during menstrual periods but also has an effect on psychological changes, such as pain perception, trait empathy, anxiety, depression, etc. [10,11,12]. In addition, some studies even showed the altered white matter microarchitecture and altered posterior cerebellar lobule connectivity with the perigenual anterior cingulate cortex [13,14,15].

In recent years, many studies have been carried out focusing on the pathological mechanism of PD and tried to develop more drugs for PD treatment [16,17,18,19,20,21,22]. One acknowledged mechanism is that the overproduction of uterine PGs causes myometrial hypercontractility, resulting in ischemia and hypoxia of the uterine muscle and pain [2,4]. Additionally, elevated vasopressin and decreased progesterone are also accompanied by PD [23,24,25,26]. Nowadays, non-steroidal anti-inflammatory drugs (NSAIDs) and oral contraceptives are used to inhibit the production of PGF_2α_ [27,28]. Oxytocin receptor inhibitors, calcium channel inhibitors, and Chinese herbal medicine are also used for relieving menstrual pain [29,30,31]. Furthermore, encouragingly, there are studies which showed that some drugs and treatments had a long-term menstrual pain relief effect [32,33,34].

Though many drugs are used for menstrual pain in the clinic, most of them have common shortcomings of periodic medication and inevitable side effects [18]. Furthermore, due to the lack of an appropriate animal model, PD-related studies were only conducted on acute animal models or clinical studies [17,26,35,36], which hindered further research on PD pathological and the development of drugs for PD treatment. Unlike other animal models of chronic pain, an ideal PD model is very rare because the periodically occurring characteristics of menstrual pain are difficult to simulate [37].

Appropriate animal models, as replicas of human diseases, can recapitulate disease pathophysiology and clinical features [38]. Here, we describe a pioneering mouse model of recurrent PD induced by estrogen combined with oxytocin. This mouse model fully characterized the PD-related clinical features for two consecutive cycles. A metabolic analysis was performed in this study to validate the major features of the PD model and explore the mechanism of periodical PD attacks. The establishment of this mouse model could be used to investigate the pathological mechanism of recurrent PD and to screen radical treatment drugs for PD.

## 2. Results

### 2.1. Estrous Cycle Monitor

**Figure 1:**
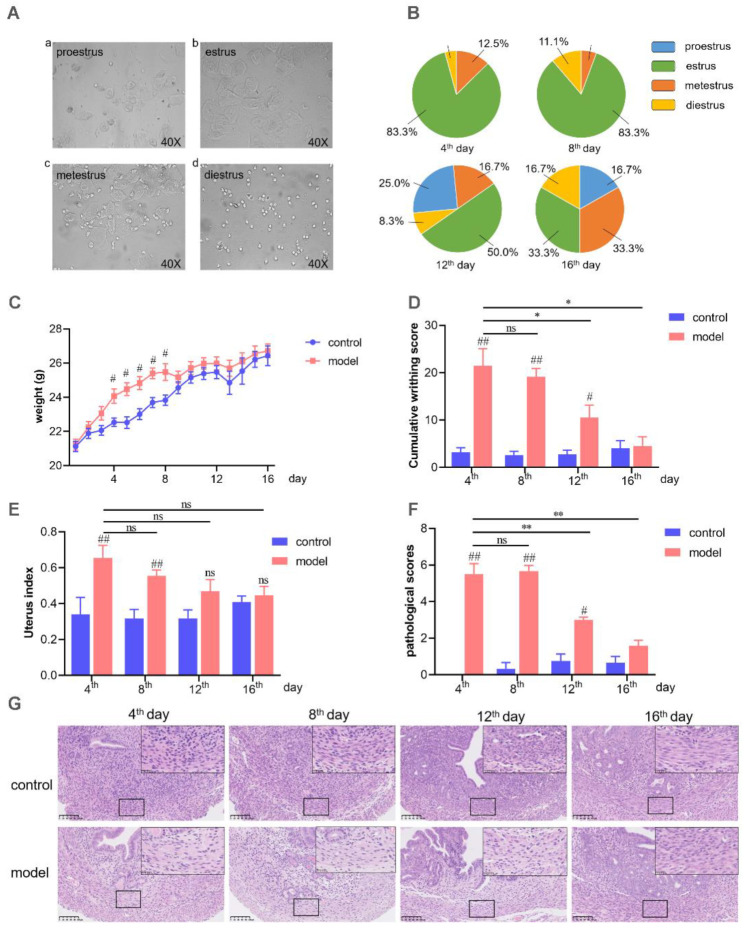
The regular detection indexes relative to the PD mice model. (**A**) Photomicrographs of vaginal smear from mice at proestrus (**a**), estrus (**b**), metestrus (**c**), and diestrus (**d**). (**B**) The percentage of each estrous stage from model group on 4th, 8th, 12th, and 16th days. (**C**) Body weight changes of the mice. (**D**) Oxytocin-induced writhing response score during the experiment. (**E**) Uterus index of control group and model group on 4th, 8th, 12th, and 16th days. (**F**) Pathological scores of uterus H&E staining. (**G**) Example pathological section of the uterus with H&E staining (20×). ^#^ Model group compared with corresponding control group. ^#^ *p* < 0.05; ^##^ *p* < 0.01. * *p* < 0.05; ** *p* < 0.01. ns, no significance. (n = 6 per group). The statistical values are presented in Table S1.

After three consecutive days of estradiol benzoate injection, 83.33% of the mice reached the estrus stage (fourth day). Interestingly, on the eighth day, vaginal smears demonstrated that 83.33% of the mice were in estrus again, but only 50% and 33.33% of them were in estrus on the 12th day and 16th day, respectively (Figure 1A,B). It suggested that the estrus cycle synchronization induced by estradiol benzoate was maintained for at least two estrous cycles (8 days).

### 2.2. Writhing Responses and Uterine Morphological Changes

Women’s menstrual cycle, which is associated with menstrual distress, is always accompanied by water retention [39,40]. Therefore, the mouse body weight was recorded daily, and the average body weight of the model mice was significantly increased compared to the control mice from the 4th day to the 8th day (Figure 1C). The writhing response was similar in mice being treated with estradiol after the 4th and 8th days but decreased in mice treated after 12 days. The writhing score of the model group was even reduced compared to the control group on the 16th day (Figure 1D). Furthermore, the uterine tissue showed edema on both the 4th day and the 8th day but no significant difference on the 12th day and 16th day between the model group and the control group (Figure 1E).

### 2.3. Histomorphology Assessment of Uterus

Severe menstrual pain induces uterine histomorphological alterations, including discontinuous smooth muscle cells and disorganized arrangement of muscle fibers, i.e., disarray [16,21]. As shown in Figure 1F,G, compared with the control group, mice in the model group showed significant pathological changes on the 4th and 8th days. However, the uterine histomorphology started to recover from the 12th day. On the 16th day, there was no significant difference between the control group and the model group.

### 2.4. Characterization of Uterine Artery Blood Flow Features

**Figure 2:**
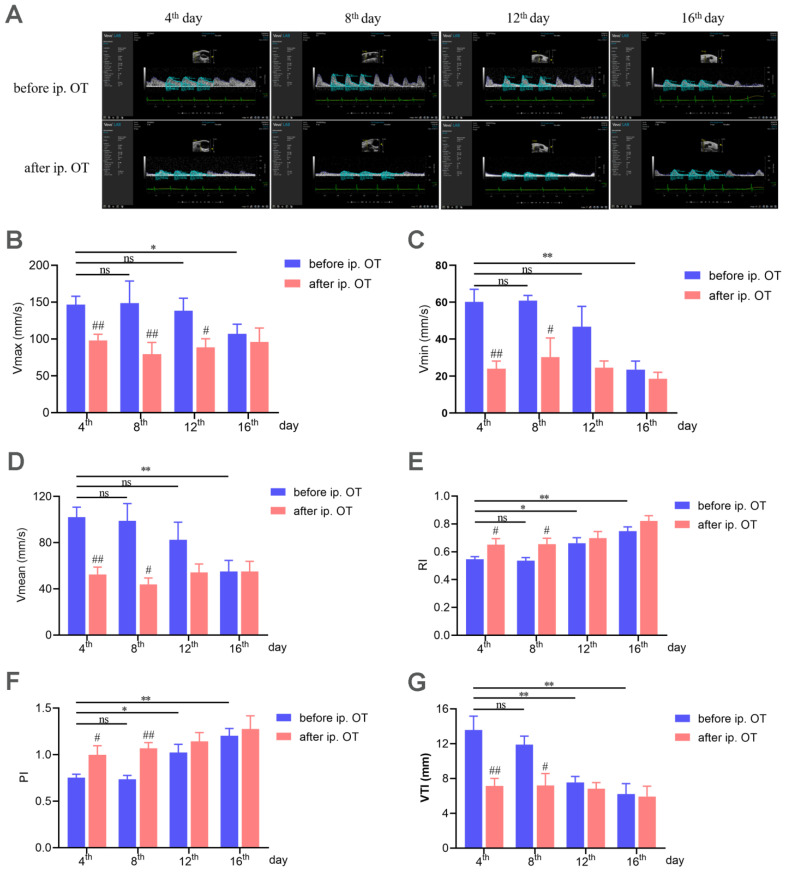
PD-related uterine artery blood flow features. (**A**) Typical images of uterine artery Doppler waveforms from model mice before and after oxytocin injection. (**B**) The maximum flow velocity (V_max_) of the uterine artery. (**C**) The minimum flow velocity (V_min_) of the uterine artery. (**D**) The average flow velocity (V_mean_) of the uterine artery. (**E**) Resistive index (RI) of uterine artery blood flow. (**F**) Pulsatility index (PI) of the uterine artery. (**G**) Velocity time integral (VTI) of uterine artery blood flow. ^#^ Model group after oxytocin injection compared with the corresponding model group before oxytocin injection. ^#^ *p* < 0.05; ^##^ *p* < 0.01. * *p* < 0.05; ** *p* < 0.01. ns, no significance. (n = 6 per group).

By evaluating writhing response and histomorphology change, we showed that the PD-related indexes were significantly affected by the oxytocin injection in model mice, while no change was observed in the control group. We compared multiple blood flow indexes in the model mice and the control group before and after oxytocin injection on the 4th, 8th, 12th, and 16th days. Figure 2A shows a representative image of the uterine artery Doppler waveform from model mice. Oxytocin injection significantly decreased the maximum, minimum, and average flow velocity (V_max_, V_min_, and V_mean_) of the uterine artery (Figure 2B–D) on the 4th and 8th days but was not able to do so on the 12th day and 16th day. The resistive index (RI) and the pulsatility index (PI) are frequently used to assess the resistance in the pulsatile vascular system. Here, after oxytocin injection, the RI was elevated on the 4th and 8th days, but no difference was detected on the 12th or 16th days (Figure 2E). Similarly, the PI was also elevated on the 4th and 8th days after oxytocin injection but was not changed on the 12th or 16th days (Figure 2F). The velocity time integral (VTI) declined on the 4th and 8th days but showed no change on the 12th or 16th days (Figure 2G). These data demonstrate that the model mice exhibited PD-induced uterine artery blood flow features on both the 4th and 8th days.

### 2.5. Biochemical Analysis of PD-Related Indicators

**Figure 3:**
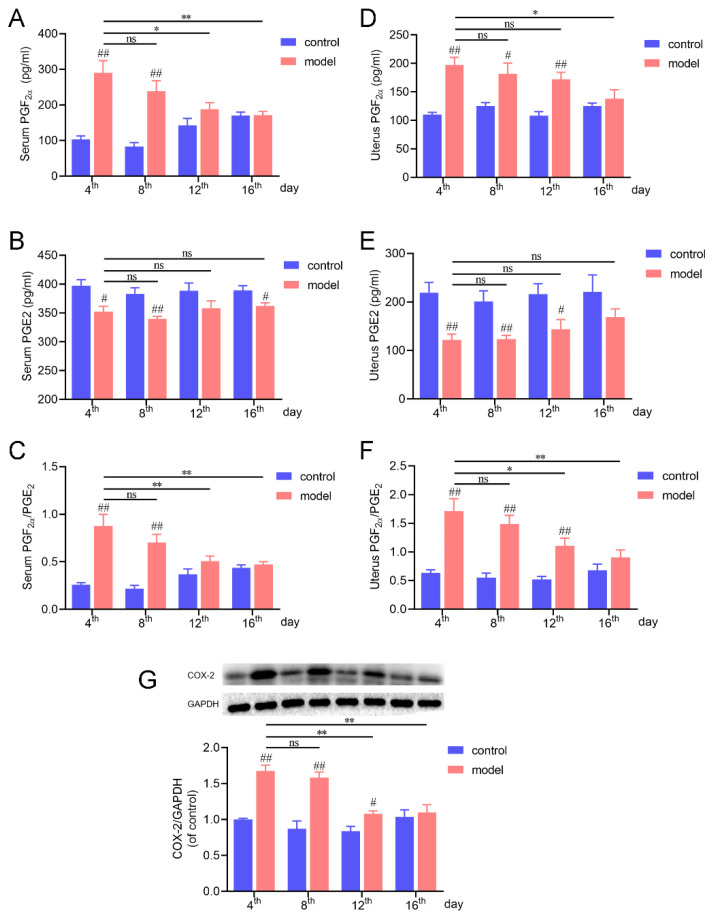
PD-related biochemical analysis. (**A**–**C**) PGF_2α_, PGE_2_, and their ratio changes in serum. (**D**–**F**) PGF_2α_, PGE_2_, and their ratio changes in uterine tissue. (**G**) COX-2 expression changes in uterine tissue during the experiment. ^#^ Model group compared with corresponding control group. ^#^ *p* < 0.05; ^##^ *p* < 0.01; * *p* < 0.05; ** *p* < 0.01. ns, no significance (n = 6 per group). The statistical values are presented in Table S1.

In the clinic and acute PD model mice, PGF_2α_ substantially increases in both serum and uterine tissue, but PGE_2_ decreases [22,23]. Here, the levels of PGF_2α_ and PGE_2_ were measured on the 4th, 8th, 12th, and 16th days. As shown in Figure 3A–F, PGF_2α_ was elevated but PGE_2_ was reduced in serum and the uterus and the ratio of PGF_2α_ and PGE_2_ was increased on both the 4th and 8th days. However, on the 12th day, a change in PGF_2α_ and PGE_2_ was only observed in uterine tissue. On the 16th day, oxytocin injection was unable to induce a change in PGF_2α_ and PGE_2_ in the serum, nor in the uterus.

### 2.6. COX-2 Expression

Cyclooxygenase 2 (COX-2), which can convert arachidonic acid (AA) into PGs, is an essential target in PD treatment [24]. Here, we showed that the expression of COX-2 was significantly upregulated on the 4th and 8th days in the model mice. The COX-2 induction was decreased from the 12th day and recovered to the basal level on the 16th day.

Our data showed that estradiol benzoate combined with oxytocin injection induced analogous menstrual pain in a similar context on the 4th and 8th days. Here, this PD mouse model is characterized as recurrent PD which was able to maintain its features for two estrous cycles.

### 2.7. Serum Metabolomics Analysis

**Figure 4:**
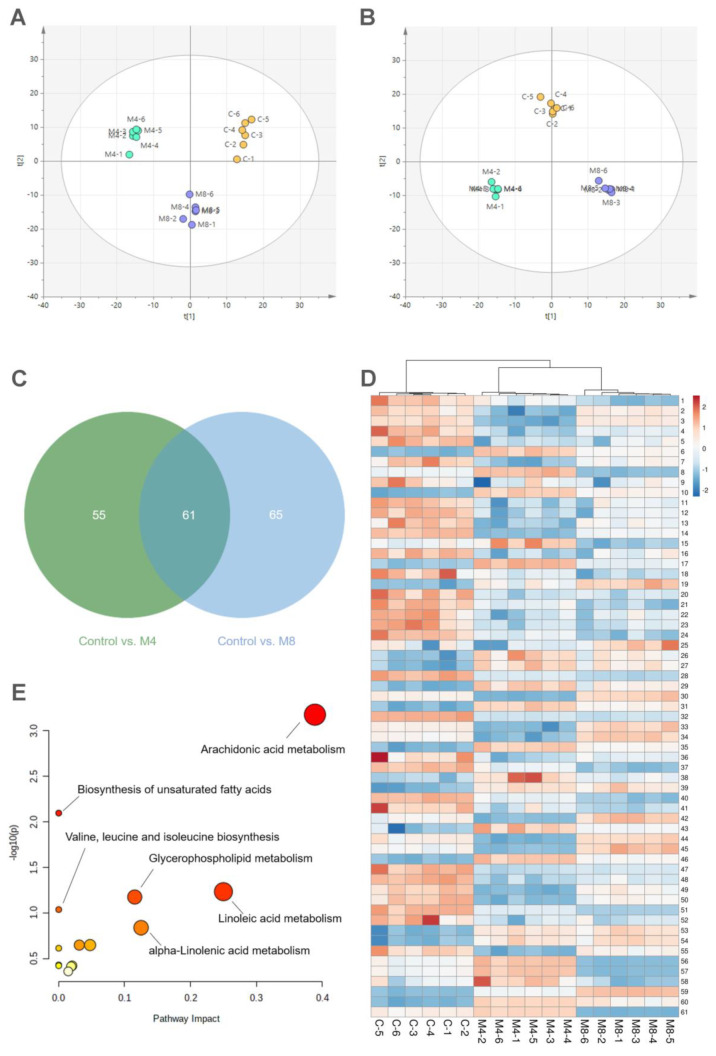
Serum metabolomics analysis of recurrent PD mouse model. (**A**) Principal component analysis (PCA) in positive mode. (**B**) PCA in negative mode. (**C**) Venn diagram of important metabolites relative to PD recurrence shared between control group vs. M4 group and control group vs. M8 group. (**D**) Heat map of important metabolites significantly changed in M4 group and M8 group compared to the control group. (**E**) Pathway analysis of important metabolites. n = 6 per group.

We next performed a serum metabolomics analysis using this recurrent PD mouse model. The model mice were euthanized on the fourth day (M4) and the eighth day (M8). A clear separation was observed between the control (C) and M4/M8 groups in the principal component analysis (PCA) with both positive ion mode and negative ion mode (Figure 4A,B). We next compared the metabolic profiles of the control group vs. the M4 group and the control group vs. the M8 group using orthogonal partial least squares discriminant analysis (OPLS-DA) (Figure S1C–F). The overlapped metabolites might be involved during PD recurrence. We found a total of 61 metabolites that were significantly changed in the M4/M8 groups compared to the control (Table S2), and the relative expression levels are shown in Figure 4D. According to the enrichment of pathways (Figure 4E), arachidonic acid metabolism; biosynthesis of unsaturated fatty acids; linoleic metabolism; glycerophospholipid metabolism; and valine, leucine, and isoleucine biosynthesis are most affected.

**Figure 5:**
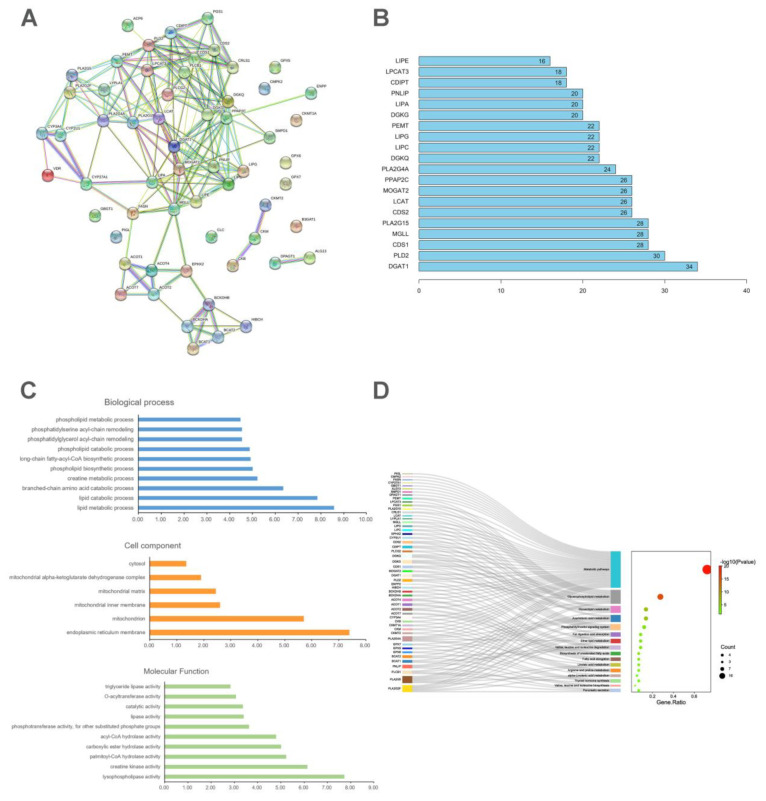
Protein–protein interactions and enrichment pathway analysis of regulatory enzymes. (**A**) Regulatory protein network map. (**B**) Top 20 proteins with high degree value in the PPI network. (**C**) GO enrichment analysis of the regulatory proteins. (**D**) KEGG enrichment analysis of the regulatory proteins.

Furthermore, we performed a pathway and enrichment analysis of related regulatory enzymes. The protein interaction network was built up and is shown in Figure 5A. The node degree was ranked and the top 20 are shown in Figure 5B. The GO enrichment analysis suggested that the changed regulatory enzymes are mainly enriched in the lipid metabolic process, lipid catabolic process, and branched-chain acid catabolic process, which might play an important role in the development of PD. In addition, these enzymes are also enriched in the endoplasmic reticulum membrane and mitochondria (Figure 5C). The KEGG enrichment analysis indicated that these regulatory enzymes are involved in metabolic pathways, glycerophospholipid metabolism, glycerolipid metabolism, arachidonic acid metabolism, the phosphatidylinositol signaling system, etc. (Figure 5).

## 3. Discussion

Primary dysmenorrhea (PD), characterized by recurrent crampy lower abdominal pain during menstruation but without pelvic pathology, is the most common complaint of women and affects more than 50% of women globally [1,4]. PD is a complex disorder involving hormone secretion, inflammation, metabolism, and sensory nerve conduction [41]. It is generally recognized that increased PGF_2α_ causes uterine contractions, which restricts blood flow and then produces hypoxia and ischemia in uterine tissue and perception of pain [1,2,4]. A clinical study showed that women with PD not only have elevated serum PGF_2α_ but also have higher serum ischemia-modified albumin (IMA) than those without menstrual pain during their menstrual period, indicating the role of ischemia in PD [42]. 

Though much PD-related research has been developed, it depends heavily on acute PD animal models or clinical trials. Animal model tests cannot achieve long-term monitoring of PD pathological processes, and clinical trials are not only time-consuming and expensive but also usually cannot meet the demand of basic research needs. Thus, developing an ideal PD animal model is quite necessary.

The animal models used at present are acute (single estrus cycle) PD models, which cannot present the characteristics of recurrent PD. Herein, we developed a mouse model of PD to mimic the periodic attacks of menstrual pain of women in the clinic. To the best of our knowledge, this is the first model of recurrent menstrual pain. As an improvement of the previously established acute PD model (one cycle, 4 days), this mouse model presented the same features of PD on the eighth day that we saw on the fourth day (Figure 1). In order to verify the consistency of PD indexes on both the 4th and 8th days, assessment of the cumulative writhing score, uterine tissue morphology, and uterine artery blood flow and biochemical analysis were performed. It is generally accepted that PD is mainly related to the abnormal synthesis and release of prostaglandins, resulting in hypoxia and ischemia of uterine muscle and the perception of pain [1,2,43]. PGF_2__α_ has a negative effect as it induces potent vasoconstriction and myometrial contractions, whereas PGE_2_ dilates blood vessels and increases blood flow. An increased ratio of PGF_2__α_/PGE_2_ leads to enhanced uterine smooth muscle contraction [44,45]. On both the 4th and 8th days, oxytocin injection induced a severe writhing response (Figure 1) and increased PGF_2α_ but reduced PGE_2_ contents in serum and the uterus; furthermore, the ratio of PGF_2__α_/PGE_2_ also increased (Figure 3). In addition, COX-2, the major enzyme that induces PGF_2α_ production [42], was also significantly upregulated on the 4th and 8th days (Figure 3). What is more, the model mice showed decreased uterine artery blood flow and increased RI and PI on the 8th and 4th days. All these parameters in our research indicated that this PD mouse model could mimic the characteristics of the recurrence of PD.

As a miniature of human diseases, animal models make it convenient for researchers to take samples at any time according to the experimental purposes, which is difficult to do in clinical trials. In this regard, this recurrent PD model increases the window for sample collection, which is helpful for pathophysiology research on PD. In addition, this model is beneficial for screening drugs with long-term efficacy to address clinical needs.

Metabolomics analysis is a comprehensive examination of various metabolite reactions and is an effective technology for biomarker discovery, disease diagnosis, and therapeutic intervention [46,47]. To better understand the pathological mechanism of periodical PD attacks, serum samples of control mice and M4/M8 mice were analyzed by metabolomics. Finally, 61 differential metabolites were identified, which might be involved in the periodical occurrence of PD. The significantly changed metabolites were enriched in arachidonic acid metabolism; linoleic acid metabolism; glycerophospholipid metabolism; valine, leucine, and isoleucine biosynthesis; alpha-linolenic acid metabolism; and biosynthesis of unsaturated fatty acids.

Arachidonic acid metabolizes through different pathways, one of which is to generate PGF_2α_ through cyclooxygenase metabolism, and cyclooxygenase is a crucial target for alleviating menstrual pain [18,28,48,49]. Glycerophospholipid, an indispensable component of cell membranes and organelle membranes, can be hydrolyzed by phospholipase A2 to generate arachidonic acid [50,51]. Glycerophospholipid metabolism disorder leads to the excessive accumulation of arachidonic acid [52,53,54]. As a result, glycerophospholipid metabolism has an effect on the arachidonic acid pathway and influences PGF_2α_ and PGE_2_ production. In addition, clinical studies have shown that reduced blood flow and an elevated level of oxidative stress were detected in PD-affected women [55,56,57]. Linoleic acid metabolism disorder is responsible for mitochondrial oxidative phosphorylation [58]. Studies showed that linoleic acid could stimulate ROS production by activating NADPH oxidase enzyme and thus disrupt mitochondrial function [59,60]. What is more, linoleic acid is the major fatty acid moiety of cardiolipin, which is a crucial component of the inner mitochondrial membrane and plays an important role in mitochondrial bioenergetics [61]. Serum cardiolipin significantly decreased in the model mice, which suggests that mitochondrial function might be impaired during menstrual pain, leading to oxidative stress. Branched-chain amino acids (BCAAs; valine, leucine, and isoleucine) are involved in a variety of biological processes, such as energy homeostasis, nutrient metabolism, and immunoreaction [62,63]. BCAA biosynthesis is related to the energy supply of mitochondria [64,65] and might take part in the energy metabolism in the uterus during PD.

Based on the above, the regulatory enzymes of these metabolites were analyzed (Figure 5). The GO enrichment analysis showed that these regulatory enzymes are mainly enriched in lipid metabolic and catabolic processes, the long-chain fatty-acyl-CoA biosynthetic process, and the branched-chain amino acid catabolic process, which is consistent with the metabolomics analysis. Furthermore, it also showed that lysophospholipase activity, creatine kinase activity, and palmitoyl-CoA hydrolase activity might participate in the pathological process of PD. Lysophospholipase, as the regulatory enzyme of lysophospholipids, mediates lipid homeostasis and lysophospholipid signaling [50,66]. Additionally, the regulatory enzymes of differentially expressed metabolites are also enriched in the cell components of reticulum membrane and mitochondria. As mentioned above, many metabolites are relevant to mitochondrial function, so it can be speculated that mitochondria are an important target in PD research. Studies also showed that intracellular calcium was significantly increased in uterine smooth muscles after oxytocin stimulation [67,68]. Endoplasmic reticulum membrane is essential for intracellular calcium homeostasis, and here, it might contribute to PD development through regulating intracellular calcium [69,70]. Consistently with this, the KEGG pathway enrichment analysis showed that these regulatory enzymes were included in glycerophospholipid metabolism, glycerolipid metabolism, arachidonic acid metabolism, etc. These metabolites and their corresponding regulatory enzymes might play important roles in PD recurrence. This study has provided a basis for subsequent research, especially the investigation of the mechanism by which the recurrence of PD is induced.

## 4. Conclusions

In summary, we established a mouse model of recurrent PD, whose characteristics were maintained for two estrous cycles. This model can provide an excellent platform for drug screening and pathological mechanism exploration of PD. Metabolism analysis is a great tool to help find important metabolites and pathways related to the recurrence of PD for further research and verification.

## 5. Materials and Methods

### 5.1. Animals

Female non-pregnant ICR mice (20 ± 2 g weight) were provided by the Experimental Animal Center of Yangzhou University, China (Animal quality certificate number: SCXK 2017–0007). Animals were housed in a 12-h light/dark cycle room at 23 ± 1 °C with adequate food and water. All animal welfare and experimental procedures were performed according to the National Institutes of Health Guide for the Care and Use of Laboratory Animals. Experiment protocols were approved by the Animal Ethics Committee of China Pharmaceutical University.

### 5.2. Reagents

Estradiol benzoate injections and oxytocin injections were purchased from Ningbo second hormone factory (China). PGF_2α_ and PGE_2_ ELISA kits were obtained from Nanjing SenBeiJia Biological Technology Co., Ltd. (China). Bicinchoninic acid (BCA) assay kit and QuichBlockTM Western Bocking Buffer were purchased from Beyotime Biotechnology (China). COX-2 and GAPDH primary antibodies were obtained from Proteintech Group, Rosemont, IL, USA. Acetonitrile, methanol, and formic acid were obtained from Merck (Darmstadt, Germany). Ultrapure water was obtained using a Milli-Q purification system (Milford, MA, USA).

### 5.3. Animal Treatment

Forty-eight female mice were divided into a control group and a model group (24 per group). The estrous stage was monitored by vaginal smear for a total of 16 days (4 cycles) [71]. Model mice were intraperitoneally injected with estradiol benzoate (10 mg·kg^−1^·day^−1^) for three consecutive days [41]. On the 4th, 8th, 12th, and 16th days, each mouse from the experimental group was intraperitoneally injected with oxytocin (0.4 U). The cumulative writhing score was recorded for each mouse within 30 min right after the injection. Mice in the control group were intraperitoneally injected with saline from the 1st day to the 3rd day. The criteria used for scoring the writhing were described in a previous study [72]. In brief, writhing was scored from 0 to 3, where 0 refers to normal body position and behaviors; 1 refers to body leaning to left or right; 2 refers to stretching of the hindlimbs and dorsiflexion of the hind paws, body stretched and flat on the bottom, the pelvis rotated sideward; and 3 refers to abdominal muscle contraction followed by body stretching and hind limbs extension.

### 5.4. Hematoxylin–Eosin Staining of Uterine Tissue

Uterine tissue was isolated and fixed in 4% paraformaldehyde for more than 24 h right after the writhing test. After dehydration, paraffin-embedded tissues were sliced to a thickness of 4 μm, hematoxylin and eosin staining were performed as previously described [35,73].

### 5.5. Uterine Artery Blood Flow Analysis

After anesthetization with 2% isoflurane, the uterine artery blood flow of the mice in the model group was monitored using Doppler ultrasound detection (Ultrasound biomicroscope, Vevo2100TM, VisualSonics, Toronto, ON, Canada) before and after oxytocin injection. Monitoring indicators included uterine artery blood flow maximum velocity (Vmax), uterine artery blood flow minimum velocity (Vmin), and average blood flow velocity (Vmean). The pulsatility index (PI) was calculated as follows: 2x (Vmax − Vmin)/(Vmax + Vmin); the resistance index (RI) was calculated as follows: (Vmax − Vmin)/Vmax.

### 5.6. PGF_2α_ and PGE_2_ Measurement

After the writhing reaction, blood samples were collected and serum samples were obtained by centrifuging at 1000× *g* for 10 min. The uterine tissue was homogenized with PBS and then centrifuged at 15,000× *g* for 15 min to obtain the supernatant. The PGF_2α_ and PGE_2_ contents in the serum and uterine tissue were tested using ELISA kits.

### 5.7. Western Blot Analysis

Total protein of uterine tissue was extracted using radioimmunoprecipitation assay buffer, and the protein concentration was quantified using the BCA assay kit. SDS-PAGE gels and PVDF membranes were used. The blots were blocked with QuichBlockTM Western Bocking Buffer (Beyotime Biotechnology, P0252), probed with COX-2 and GAPDH primary antibodies (Proteintech Group, 66351-1-Ig and 60004-1-Ig) overnight at 4 °C, and then incubated with the appropriate secondary antibodies. The values were expressed relative to the signal of GAPDH.

### 5.8. Untargeted Metabolomics Analysis

#### 5.8.1. Serum Sample Pretreatment

At the end of each writhing experiment, serum was collected and stored at −80 °C for further metabolomics analysis. In brief, serum samples were thawed at room temperature and mixed with methanol in a ratio of 1:3. Next, the samples were centrifuged at 15,000× *g* for 10 min (4 °C), and the supernatant was transferred to a new tube. Subsequently, the supernatant was dried with nitrogen at room temperature and redissolved with methanol for further analysis.

#### 5.8.2. LC-MS Analysis

Serum metabolite separation was performed on a SynergiTM Fusion-RP C18 column (50 mm × 2 mm internal diameter (i.d.), 2.5 μm) using an Agilent Technologies 6530 Accurate-Mass Q-TOF LC/MS system (Santa Clara, CA, USA). The mobile phase consisted of solvent A (0.1% aqueous formic acid solution) and solvent B (0.1% acetonitrile formic acid). The chromatographic conditions were as follows: 0–2 min, 0–5% B; 2–10 min, 5–60% B; 10–15 min, 60–70% B; 15–20 min, 70–80% B; 20–22 min, 80–95% B; 22–28 min, 95–60% B; 28–30 min, 60–30% B, and back to initial conditions (with 2 min for equilibration). The flow rate was 0.4 mL/min. Each sample injection volume was 5 μL. The MS was performed with an electrospray ionization (ESI) ion source in the positive (ESI+) and negative (ESI−) ion modes. The data were collected in both positive and negative ion modes with a mass range of 50–2000 Da. The MS parameters were set as follows: fragmental voltage, 120 V; nebulizer gas, 35 psig; capillary voltage, 4000 V; drying gas flow rate, 9 L/min; temperature, 325 °C.

#### 5.8.3. Method Assessment

The reproducibility and robustness of our experiment were tested using a quality control (QC) sample. In total, 10 μL of each serum sample was mixed and pretreated in the same way as the test samples to obtain a QC sample. During the analysis, the QC sample was analyzed every 6 injections. Next, the retention time and intensity of 5 different ions were extracted for calculating relative standard deviations (RSDs) to verify the repeatability of the method [44,53]. The typical TICs are shown in Figure S1A,B, and the RSD results of intensity and retention time are listed in Table S3.

#### 5.8.4. Data Processing

The raw data were pretreated using an R package, including nonlinear retention time alignment, peak discrimination, filtering, alignment, and matching, etc. After that, the processed data were analyzed using SIMCA-P 14.1 (Umetrics, Umeå, Sweden) and MetaboAnalyst 3.0 on 22 November 2021 (https://www.metaboanalyst.ca/). Principal components analysis (PCA) and supervised orthogonal partial least squares discriminant analysis (OPLS-DA) were applied to analyze global metabolic profiles and detect the variables. The differential metabolites were identified with *p* < 0.05 (Student’s *t*-test) and VIP > 1.0.

#### 5.8.5. Metabolite Identification and Pathway Analysis

Differential metabolites’ identification was reliant on online databases, including HMDB (http://www.hmdb.ca/ accessed on 22 April 2022), METLIN (http://metlin.scripps.edu/ accessed on 22 April 2022), and MassBank (http://www.massbank.jp/ accessed on 22 April 2022). Pathway enrichment analysis was carried out using MetaboAnalyst. The regulated enzymes were analyzed by STRING (https://cn.string-db.org/ accessed on 22 April 2022), DAVID (https://david.ncifcrf.gov/ accessed on 22 April 2022), and bioinformatics (http://www.bioinformatics.com.cn/en accessed on 22 April 2022).

### 5.9. Statistical Analysis

The experimental data were statistically analyzed using GraphPad Prism 7. The data were analyzed by Student’s *t*-test or ANOVA and presented as mean ± SEM. A *p*-value < 0.05 was considered statistically significant.

## Data Availability

Not applicable.

## Supplementary Materials

The following supporting information can be downloaded at: https://www.mdpi.com/article/10.3390/ijms23116128/s1.
