# Supplementary material for: The Establishment of a Mouse Model of Recurrent Primary Dysmenorrhea
Source: Int J Mol Sci. 2022 May 30;23(11):6128. doi: 10.3390/ijms23116128 (PMC9181441; doi:10.3390/ijms23116128)
Supplement: Supplementary file 1 [file ijms-23-06128-s001.zip › Table S1.pdf]

Table S1. The statistical values of PD-related features.

	4th day		8th day		12th day		16th day	
	control	model	control	model	control	model	control	model
Cumulative writhing score	3.208±0.943	21.476±3.518**	2.556±0.836	19.167±1.671**	2.75±0.851	10.500±2.554*	4.000±1.491	4.500±1.791 n.s.
Uterus index	0.340±0.087	0.654±0.065**	0.317±0.046	0.554±0.031**	0.319±0.041	0.470±0.059 n.s.	0.409±0.031	0.447±0.043 n.s.
Pathological scores	0±0	5.5±0.471**	0.333±0.272	5.667±0.245**	0.750±0.312	3±0.118*	0.667±0.272	1.583±0.245 n.s.
serum $PGF_{2\alpha}$ (pg/ml)	103.137±9.051	290.114±31.525**	83.137±10.363	238.578±27.095**	142.5095±17.64	188.039±16.527 n.s.	169.902±9.327	171.029±10.201 n.s.
Serum PGE_2 (pg/ml)	397.227±9.508	352.218±8.221*	383.167±9.475	339.894±3.640**	388.545±12.123	358.272±11.563 n.s.	389.182±7.270	362.291±4.744*
Serum $PGF_{2\alpha}/PGE_2$	0.259±0.020	0.878±0.099**	0.219±0.030	0.701±0.079**	0.370±0.044	0.507±0.047 n.s.	0.437±0.025	0.471±0.026 n.s.
uterus $PGF_{2\alpha}$(pg/ml)	110.099±3.524	197.333±12.043**	125.259±5.587	181.679±17.336*	107.951±6.784	172.099±11.307**	125.155±4.882	137.985±14.272 n.s.
uterus PGE_2 (pg/ml)	219.137±19.141	121.756±10.742**	200.893±20.057	123.244±7.196**	216.041±19.615	144.226±17.947*	221.131±39.392	169.107±15.094 n.s.
Uterus $PGF_{2\alpha}/PGE_2$	0.635±0.049	1.712±0.178**	0.550±0.074	1.489±0.136**	0.519±0.050	1.103±0.114**	0.677±0.102	0.903±0.120 n.s.

*, model group compared with corresponding control group. * $p<0.05$, ** $p<0.01$.
