# Supplementary material for: The Establishment of a Mouse Model of Recurrent Primary Dysmenorrhea
Source: Int J Mol Sci. 2022 May 30;23(11):6128. doi: 10.3390/ijms23116128 (PMC9181441; doi:10.3390/ijms23116128)
Supplement: Supplementary file 1 [file ijms-23-06128-s001.zip › Table S2.pdf]

Table S2. Identification of sharing differential metabolites in serum.

	No	m/z	RT (min)	Metabolites	HMDB ID	M4 vs. Control			M8 vs. Control			
						p-value	VIP	FC	*p*-value	VIP	FC	
positive	1	519.6515	17.91	LysoPC(18:2(9Z,12Z)/0:0)	HMDB10386	<0.001	4.76	0.92	<0.001	9.29	0.85	LYPLA1, PLA2G15, PLA2G5
	2	543.6729	17.87	LysoPC(20:4(8Z,11Z,14Z,17Z)/0:0)	HMDB0010396	<0.001	4.18	0.89	0.045	1.69	0.98	LYPLA1, PLA2G15, PLA2G5
	3	521.6673	18.68	LysoPC(18:1(11Z)/0:0)	HMDB0010385	<0.001	6.52	0.73	0.002	2.90	0.96	LYPLA1, PLA2G15, PLA2G5
	4	987.589	17.87	Triacylglycerol	HMDB0049505	<0.001	3.17	0.80	<0.001	3.41	0.86	PNLIP, LIPC, LIPA, LIPG, DGAT1
	5	675.092	26.3	Diacylglycerol	HMDB0056222	<0.001	3.40	0.30	<0.001	3.68	0.48	DGKQ, PNLIP, PLCB1, DGKG
	6	567.3324	17.91	LysoPC(22:6)	HMDB0010404	<0.001	4.65	1.28	<0.001	4.76	1.17	LYPLA1, PLA2G15, PLA2G5
	7	507.6838	18.09	LysoPC(P-18:0/0:0)	HMDB0013122	<0.001	1.37	0.88	0.002	1.06	0.95	PLA2G4A, LCAT, CLC, LPCAT3
	8	396.6484	9.19	Ergocalciferol	HMDB0000900	<0.001	2.55	1.27	<0.001	3.16	0.76	CYP3A4, CYP27A1, VDR, CMPK2
	9	338.4816	26.3	8,9-DiHETrE	HMDB0002311	0.048	2.52	0.77	0.028	3.33	0.79	EPHX2
	10	525.6145	17.62	LysoPE(22:6(4Z,7Z,10Z,13Z,16Z,19Z)/0:0)	HMDB0011526	<0.001	3.46	1.61	<0.001	3.74	1.41	ENPP2
	11	1391.919	14.71	Cardiolipin(67:2)	HMDB0080408	<0.001	1.44	0.91	<0.001	1.46	0.94	CRLS1
	12	706.942	14.71	Phosphatidate(37:6)	HMDB0115403	<0.001	1.62	0.90	<0.001	1.77	0.93	PLD2, DGKQ, PPAP2C
	13	941.52	18.63	TG(22:6(4Z,7Z,10Z,13Z,16Z,19Z)/20:4(5Z,8Z,11Z,1	HMDB0055932	<0.001	2.88	0.52	0.001	2.35	0.78	PNLIP, LIPC, LIPA,

			4Z)/O-18:0)								LIPG
14	204.2252	1.02	(±)-Tryptophan	HMDB0030396	<0.001	2.31	0.71	<0.001	2.14	0.85	-
15	256.254	19.17	3-hydroxy-3-(2,3,4-trimethoxyphenyl)propanoic acid	HMDB0142079	0.001	1.42	1.34	<0.001	1.86	0.69	-
16	1162.22	14.71	3-Hydroxyhexacosanoyl-CoA	HMDB0062351	0.002	1.48	0.88	<0.001	1.48	0.92	-
17	302.3649	18.76	Enterodiol	HMDB0005056	<0.001	1.70	1.33	<0.001	1.32	1.12	-
18	782.0817	28.78	PC(18:2(9Z,12Z)/18:2(9Z,12Z))	HMDB0008138	0.001	1.34	0.62	<0.001	2.25	0.44	LYPLA1, PLA2G15, PLA2G5
19	1111.694	17.88	-	-	<0.001	1.07	1.10	<0.001	1.92	1.18	-
20	545.348	18.34	LysoPC(20:3)	HMDB0010393	<0.001	1.34	0.84	<0.001	1.69	0.85	LYPLA1, PLA2G15, PLA2G5
21	1089.18	14.71	-	-	<0.001	1.35	0.85	<0.001	2.24	0.76	-
22	1089.304	14.71	-	-	<0.001	1.16	0.88	<0.001	1.31	0.91	-
23	1089.429	14.71	-	-	<0.001	1.24	0.87	<0.001	1.59	0.88	-
24	1089.548	14.71	-	-	<0.001	1.11	0.89	<0.001	1.83	0.84	-
25	1415.941	14.92	CL(i-12:0/18:2(9Z,11Z)/18:2(9Z,11Z)/i-21:0)	HMDB0080752	<0.001	2.20	0.50	<0.001	2.52	1.39	CRLS1
26	1047.088	14.59	Phosphatidylinositol diphosphate(38:4)	HMDB0010070	<0.001	1.26	1.20	<0.001	1.29	1.13	PLCB1, PLCG2, PIGL
27	1050.174	14.59	CDP-DG(40:8)	HMDB0116007	<0.001	1.43	1.26	<0.001	1.62	1.20	CDS2
28	211.1131	0.76	Phosphocreatine	HMDB0001511	<0.001	1.59	0.65	<0.001	2.32	0.57	CKMT2, CKB, CKMT1A, CKM
29	1008.178	14.59	CDP-DG(36:1)	HMDB0006987	<0.001	1.49	1.31	<0.001	1.59	1.21	CDS2, CDS1, CDIPT, PGS1
30	1490.108	14.92	Cardiolipin(74:2)	HMDB0081537	<0.001	2.15	0.48	<0.001	1.67	1.20	CRLS1
31	328.4883	22.04	Docosahexaenoic acid	HMDB0002183	<0.001	1.15	1.48	<0.001	1.36	1.33	ACOT7, ACOT2, ACOT4, CYP2U1, ACOT1

	32	537.3794	21.2	LysoPE(0:0/22:0)	HMDB0011490	<0.001	1.40	0.69	<0.001	1.25	0.68	ENPP2
	33	1396.488	14.88	nLc6Cer	HMDB0062507	<0.001	1.57	0.65	<0.001	1.10	1.11	-
	34	1475.996	14.88	CL(18:2(9Z,12Z)/16:0/22:5(4Z,7Z,10Z,13Z,16Z)/18:2(9Z,12Z))	HMDB0058708	<0.001	1.37	0.63	<0.001	1.31	1.20	CRLS1
negative	35	328.4883	22.08	Docosahexaenoic acid	HMDB0002183	<0.001	5.05	1.44	<0.001	4.12	1.30	ACOT7, ACOT2, ACOT4, CYP2U1, ACOT1
	36	256.4241	23.56	Palmitic acid	HMDB0000220	<0.001	1.78	0.87	0.004	1.42	0.91	FASN, LYPLA1, PLA2G5
	37	565.763	17.95	LysoPE(24:0)	HMDB0011527	<0.001	4.71	0.91	<0.001	6.67	0.85	ENPP2
	38	587.5345	27.9	N-Acetyl-D-glucosaminyldiphosphodolichol	HMDB0001445	<0.001	2.26	3.67	0.001	1.26	2.03	DPAGT1, ALG13
	39	592.946	17.96	Diacylglycerol	HMDB0056138	<0.001	3.84	1.30	<0.001	3.84	1.29	DGKQ, PNLIP, PLCB1, LIPA
	40	1101.384	17.95	Cardiolipin(42:0)	HMDB0116973	<0.001	2.02	0.86	<0.001	2.68	0.78	-
	41	282.468	24.06	Octadec-9-enoic Acid	HMDB0062703	<0.001	1.62	0.90	<0.001	2.05	0.87	-
	42	1091.742	20.1	-	-	<0.001	3.11	0.63	<0.001	2.49	1.16	-
	43	320.4663	18.8	5-HETE	HMDB0011134	<0.001	3.93	1.24	0.001	2.74	1.13	GPX7, GPX5, GPX6
	44	304.4669	22.29	Arachidonic acid	HMDB0001043	<0.001	2.93	0.74	0.005	1.33	1.02	FASN, PLA2G5
	45	592.946	20.1	Diglyceride	HMDB0056157	<0.001	1.39	0.82	<0.001	1.24	1.09	DGKQ, PNLIP, PLCB1
	46	643.006	22.08	Diacylglycerol(38:5)	HMDB0056315	<0.001	1.67	1.75	<0.001	1.30	1.47	DGKQ, PNLIP, PLCB1
	47	254.2823	21.66	Aldosine	HMDB0037817	<0.001	1.52	0.75	<0.001	1.47	0.77	-
	48	1107.677	17.91	-	-	<0.001	2.06	0.84	<0.001	1.84	0.87	-
	49	521.608	18.71	Morphiceptin	HMDB0005777	<0.001	1.82	0.55	<0.001	1.01	0.81	-
	50	503.609	17.54	LysoPE(20:3)	HMDB0011516	<0.001	1.92	0.76	<0.001	1.17	0.88	ENPP2

51	378.5454	16.61	b-Monoacylglycerol	HMDB0004666	<0.001	1.02	0.45	<0.001	1.24	0.27	MGLL, LIPE, MOGAT2, ACP6
52	344.4877	24.03	16(17)-EpDPE	HMDB0013621	<0.001	1.18	0.76	<0.001	1.15	0.78	-
53	555.6227	17.66	Enkephalin L	HMDB0001045	<0.001	1.06	1.59	<0.001	1.11	1.61	-
54	1480.113	18.54	Cardiolipin(68:0)	HMDB0124210	<0.001	3.50	1.61	<0.001	3.73	1.64	-
55	90.0779	0.64	Hydroxypropionic acid	HMDB0000700	<0.001	2.15	0.81	0.004	1.09	0.92	HIBCH
56	130.1418	1.71	Ketoleucine	HMDB0000695	<0.001	2.36	1.28	<0.001	2.62	0.76	BCKDH8, BCKDHA, BCAT1, BCAT2
57	764.0664	22.08	PC(36:6)	HMDB0011280	<0.001	2.31	1.26	<0.001	2.62	0.76	PLA2G5, PLA2G2F, LCAT
58	768.07	12.56	dimethylphosphatidylethanolamine	HMDB0114046	0.005	1.42	1.10	0.01	1.20	0.95	PEMT
59	362.44	24.07	[(5-oxo-1,7-diphenylheptan-3-yl)oxy]sulfonic acid	HMDB0134658	<0.001	1.03	2.40	<0.001	1.49	3.57	-
60	1031.249	14.79	Cardiolipin(38:0)	HMDB0116873	<0.001	1.32	1.89	<0.001	1.16	1.68	-
61	676.9908	29.12	Sphingomyelin	HMDB0012085	<0.001	1.34	1.26	<0.001	1.16	0.42	GBGT1, PIGL, SMPD1, B3GAT1
