# Supplementary material for: The Establishment of a Mouse Model of Recurrent Primary Dysmenorrhea
Source: Int J Mol Sci. 2022 May 30;23(11):6128. doi: 10.3390/ijms23116128 (PMC9181441; doi:10.3390/ijms23116128)
Supplement: Supplementary file 1 [file ijms-23-06128-s001.zip › Table S3.pdf]

Table S3. RSD of typical peaks of QC sample（ESI^+, n=10; ESI^-, n=10).

Ion Mode	MS	RSD (%)	
		Retention Time	Peak Intensity
positive	104.1086	1.2110	0.9164
	274.2775	0.0269	0.4353
	520.3501	0.0054	0.4324
	496.3495	0.0089	0.3436
	524.3811	0.0048	0.8755
negative	89.023	0.3924	0.2353
	564.3254	0.0161	0.8796
	540.3253	0.0162	1.0242
	568.3566	0.0143	1.6701
	281.2451	0.0124	1.2774
