# Supplementary material for: The Establishment of a Mouse Model of Recurrent Primary Dysmenorrhea
Source: Int J Mol Sci. 2022 May 30;23(11):6128. doi: 10.3390/ijms23116128 (PMC9181441; doi:10.3390/ijms23116128)
Supplement: Supplementary file 1 [file Fig.S1.pdf › Fig.S1.pdf]

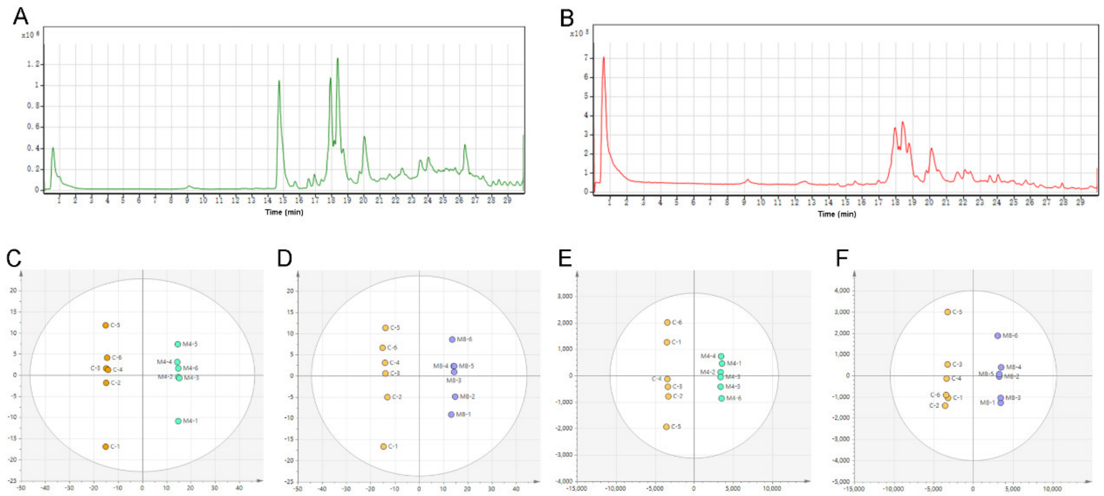

Figure S1. TICs and score plots from serum metabolomics analysis. (**A**) Typical positive TIC of QC sample. (**B**) Typical negative TIC of QC sample. (**C**,**D**) OPLS-DA score of control vs. M4 and control vs. M8 (positive mode). (**E**,**F**) OPLS-DA score of control vs. M4 and control vs. M8 (negative mode). (n = 6 per group).
